# Genome-wide association study identifies key quantitative trait loci (QTL) for fruit morphometric traits in avocado (*Persea* spp.)

**DOI:** 10.1186/s12864-024-11043-1

**Published:** 2024-11-25

**Authors:** Jin Li, Shamseldeen Eltaher, Barbie Freeman, Sukhwinder Singh, Gul Shad Ali

**Affiliations:** 1 United States Department of Agriculture, Agricultural Research Service, Subtropical Horticulture Research Station, Miami, FL USA; 2 Present Address: Department of Plant Biotechnology, Genetic Engineering and Biotechnology Research Institute (GEBRI), University of Sadat City, Sadat, Egypt

**Keywords:** Avocado, Fruit morphometric traits, Phenotypic variation, Chip-based genotyping, Genetic diversity, Population structure, Genetic mapping, Genome-wide association study (GWAS)

## Abstract

**Background:**

Avocado, a fruit crop renowned for its high nutritional value, has seen a steadily increasing market demand. However, breeding efforts for avocados have lagged those for other commercial fruits due to limited genomic research and germplasm resources. To address this shortfall, a genome-wide association study was conducted on 122 avocado accessions from the United States Department of Agriculture (USDA) Agricultural Research Service (ARS) Subtropical Horticultural Research Station (SHRS) germplasm collection. The study aimed to identify genetic markers and regions associated with various morphometric traits in avocado fruits, including fruit weight, length, width, diameter, seed weight, seed length, seed width, fruit seed ratio (FSR), and fruit shape index (FSI).

**Results:**

Leveraging 4,226 high-quality single nucleotide polymorphism (SNP) markers obtained from genotyping arrays, fifteen markers were identified with strong associations with these traits, utilizing Bayesian-information and Linkage-disequilibrium Iteratively Nested Keyway (BLINK) and Fixed and random model Circulating Probability Unification (FarmCPU) models. Gene annotation analyses within a 200-kb window in the vicinity of significant SNPs revealed several genes associated with various metabolic pathways suggesting that some of them likely determine these fruit quality traits, which needs to be verified and validated. Our results, which were conducted at one location, provide directions for conducting future studies using high-resolution genotyping and long-term multi-year and multi-location trait evaluations for precisely identifying the causal SNP(s) and genes associated with these traits.

**Conclusions:**

These markers reported in this manuscript provide valuable tools for applying marker-assisted selection (MAS) in avocado breeding programs aimed at enhancing fruit quality and value.

**Supplementary Information:**

The online version contains supplementary material available at 10.1186/s12864-024-11043-1.

## Background

Avocado (*Persea americana*) is a tropical fruit crop known for its high nutritional value and economic importance. Rich in monounsaturated fats and various vitamins, avocado’s global popularity has been increasing over the years due to the rising demand for healthy foods and alternative dietary fat sources [1]. Currently, avocados are produced in more than 57 countries, with Mexico, Colombia, Peru, Dominican Republic, Kenya, Indonesia, and Brazil among the top producing countries. Mexico is the leading producer, accounting for over 28% of world production [2]. In only a decade, avocado production doubled from 4.41 million tons in 2012 to 8.98 million tons in 2022 [3]. In 2023, avocado exports from Mexico alone amounted to nearly 2.84 billion U.S. dollars [4].

Avocado belongs to the genus *Persea* within the Lauraceae family. The genus *Persea* is divided into two subgenera: *Persea* (avocado) and *Eriodaphne* (avocado-like). The subgenus *Persea*, which has commercial significance, contains three botanical races based on their geoclimatic origins: *P. americana* var. *drymifolia* (Mexican race), *P. americana* var. *guatemalensis* (Guatemalan race), *P. americana* var. *americana* (West Indian race) [5]. These classifications are supported by molecular genetic data [6]. Mesoamerica is considered the center of the origin of avocados, and it still harbors genetic diversity in the wild that is at risk due to climate change [7, 8].

Approximately 80% of global avocado cultivation is of the ‘Hass’ cultivar, which is suggested to be a hybrid of Mexican and Guatemalan ecotypes [8, 9]. Although ‘Hass’ is highly heterozygous, the predominance of a single cultivar reflects a very narrow genetic background in commercial avocado farming. However, the genetic background of different avocado ecotypes is quite diverse, with each group displaying variability in fruit quality and horticultural traits [9, 10]. These ecotypes appear to have hybridized only recently, and each potentially carries advantageous alleles in unique combinations [11]. The USDA ARS SHRS maintains a collection of 167 avocado accessions representing the three ecotypes and hybrids collected worldwide [12], providing valuable resources for germplasm genetic diversity and breeding studies.

Leveraging next-generation and advanced sequencing technologies, the avocado genome has been sequenced, revealing a total chromosome number of 2n = 24 and a haploid genome size of approximately 920 Mb [9, 13]. Together with technologies like genotyping-by-sequencing (GBS) and chip-based genotyping, these advancements facilitate breeding efforts such as Marker-Assisted Selection (MAS) by providing a detailed map of genetic variants, including single nucleotide polymorphisms (SNPs) [14]. Based on this data, Genome-Wide Association Studies (GWAS) can be performed to identify associations between genotypes and specific phenotypes of interest [15, 16]. This enables the efficient selection of parent plants with desirable traits through rapid genotyping, rather than relying solely on the traditional observation of phenotypes, which can be influenced by environmental factors. Traditional methods are time-consuming, especially for fruit trees like avocados that have a long juvenile period of up to 15 years before flowering and producing fruit [17]. This lengthy period of development significantly hinders the breeding process.

Fruit quality traits such as fruit weight and size are pivotal for breeding as they correlate with higher yield, improved nutritional content, and consumer preference, thereby increasing profitability for farmers and producers [18, 19]. Investigating traits like fruit size, however, is complex, involving multiple factors, and no single growth regulator can fully explain the entire process of fruit morphogenesis [20–22]. Instead, a combination of growth regulators and environmental factors collectively shape the process [23]. Genome-wide association studies (GWAS) have effectively studied fruit quality traits including soluble solid content, sugars, titrable acidity, and fruit size, weight, shape, texture, skin color, flesh color, and seedlessness in various fruit crops such as grapes, apples, peaches, apricots, plums, cherries, chestnuts and citruses [24]. For example, a study on 355 apple genotypes identified 59 SNP markers associated with apple fruit size and shape, with the most significant markers indicating that genes from the ovate family play a crucial role in determining fruit size in apple [25]. Another study on 312 sand pear (*Pyrus pyrifolia*) accessions identified 37 SNP markers for 8 fruit quality traits, revealing the gene *PbrSTONE* related to stone cell lignification and its role in regulating stone cell formation in pears [26]. Despite these, few studies have explored fruit quality traits in avocados.

A genotyping chip comprising 5,050 high-quality SNP markers, formulated using transcriptome data from prominent avocado cultivars (‘Hass,’ ‘Bacon,’ ‘Simmonds’ and ‘Tonnage’), was developed as per Kuhn et al.’s work in 2019 [27]. A subset of these SNP markers (1,235) was selected for Quantitative Trait Locus (QTL) mapping within a population derived from a ‘Gwen’ (G) × ‘Fuerte’ (F) cross. Although with limited statistical power, this mapping successfully pinpointed a QTL for fruit β-sitosterol content in Linkage Group 1 of the Hass genome, a region in Linkage Group 3 correlating with fruit vitamin E (α-tocopherol) content, and numerous markers in Linkage Group 10 linked to avocado flowering type [28].

In this study, we mapped the 5,050 SNP markers to the most recent Hass reference genome (GCA_029852735.1) [13]. Following stringent data quality control measures, 4,226 SNP markers were retained and subsequently utilized to assess the genetic diversity of 122 selected avocado accessions cultivated in USDA ARS SHRS fields. Furthermore, GWAS was performed to identify significant genetic markers and genomic regions associated with nine key fruit quality morphometric traits, including fruit weight, length, width, diameter, seed weight, length, width, fruit shape index (FSI), and fruit seed ratio (FSR), leveraging phenotypic data collected over the period from 2015 to 2023. Potential genes and pathways underlying these traits were identified through functional inference. This work provides insights into fruit development and paves the way fore breeding elite avocado cultivars in the future.

## Methods

### Plant material and growth conditions

The experiments reported in this study were conducted at the United States Department of Agriculture, Agricultural Research Service (USDA-ARS) Subtropical Horticulture Research Station (Miami, FL). All *Persea* spp. accessions reported in this study are part of the USDA-ARS National Plant Germplasm System, which are maintained in the field at the National Germplasm Laboratory in Miami, FL. These trees, grafted on ‘Lula’ rootstocks and spaced 20 feet apart, receive fertilization in March, June, September, and December with a slow-release fertilizer (8-2-14, N-P-K). Weeds are controlled by treating the areas around the trees with herbicide (Glyphosate or Glufosinate Ammonium at the label rate) when needed. The average yearly precipitation in Miami, Florida is approximately 62.00 inches (https://www.weather.gov/mfl/climate) and therefore no irrigation is needed. To allow for natural growth, these trees are not pruned regularly, however, low branches that are too close to the ground are pruned back. Fruits from 122 accessions were collected and phenotyped between 2015 and 2023. A list of *Persea* sp. accessions utilized is provided in Supplementary Table S1. We collected 12 ripe fruits for each accession at the time of full harvest maturity based on power analysis [29], with some accessions having up to three sets of fruits. The ripeness assessment criteria included observing fruit skin darkening, crater formation at the stem-fruit junction, and softening fruit texture.

### Phenotyping of fruit morphometric trait

Nine fruit morphometric traits were measured, including fruit weight, length, width, diameter, seed weight, seed length, seed width, fruit shape index (FSI), and fruit seed ratio (FSR). Fruit and seed weights were determined using an electronic scale. Length and width were measured using calipers. To measure fruit diameter, the fruit was slid through premeasured circular wooden paddles of varying sizes. FSI was calculated as the ratio of fruit length to diameter, where a higher FSI indicates a prolonged clavate shape, while a low FSI represents an oblate fruit shape. FSR, the fruit weight to seed weight ratio, reflects the proportion of pulp content, with a high FSR indicating more pulp and a low FSR indicating less pulp in the fruit.

To normalize the data distribution, all traits were subjected to Box-Cox transformation [30] in R v. 4.3.2 [31] with MASS package [32]. Pearson correlation coefficients (*r*) between traits were calculated using the cor function in R for both the original data and Box-Cox transformed traits. The coefficient (*r*) matrix was visualized as triangle heatmaps using custom R code with ggplot2 package [33].

### DNA extraction and chip-based genotyping

DNA extraction was carried out for 122 selected avocado accessions using the FastPrep method (MPBio, Santa Ana, CA). Subsequently, the Illumina Infinium II 6000 SNP chip (Illumina, San Diego, CA, USA) was employed for genotyping, utilizing 5,050 high-quality SNPs designed based on transcriptomic data from Hass and other commercial cultivars as described in a previous study [27]. During this work, a new version of the Hass reference genome (GCA_029852735.1) [13] was released with improved completeness and continuity. The Burrow-Wheeler Aligner v. 0.7.17 [34] was used to map contigs for the SNP design to the latest Hass genome and update SNP coordinates. Furthermore, due to the initial lack of functional annotation information for the genome released in NCBI, we carried out genome annotation using eggNOG-mapper v. 2.1.12 [35] against the eggNOG v. 5.0 database [36] and annotated the 5,050 SNPs with custom Python code.

SNP data were filtered according to the following criteria to improve data quality for subsequent analyses. SNP with a Minor Allele Frequency (MAF) below 0.05 were excluded, as low MAF can reduce statistical power and cause spurious associations. Additionally, SNPs with high missingness were filtered out to prevent distorted analysis and biased results. SNPs missing in more than 20% of accessions were excluded, and the threshold for missing data was set at 5% per accession.

### Genetic diversity and population structure analyses

A comprehensive genetic analysis was conducted on 4,226 SNPs across the 122 avocado accessions to shed light on the genetic diversity and structure of the avocado germplasm. The population structure analysis was conducted using ADMIXTURE v. 1.3.0 [37], with a range of K values, spanning from 1 to 10, to ascertain the optimal number of subpopulations present with the dataset based on cross-validation errors. To assess population differentiation, an analysis of molecular variance (AMOVA) was performed with 4,226 SNP markers using GeneAlEx v.6.503 [38].

Principal component analysis (PCA) was performed using PLINK v. 2.0 [39] to calculate the top 10 principal components. The first two components were visualized as a dot plot by ggplot2 in R, to give insights into the genetic diversity and structure inherent within the avocado accessions.

To further provide insights into the genetic relationships among the accessions, a maximum-likelihood (ML) tree was constructed using RAxML v. 8.2.12 [40] with the General Time Reversible (GTR) model and the gamma distribution after aligning sequence concatenated by 4,226 SNP markers using MAFFT v. 7.505 [41]. The topology was supported by 1,000 bootstrap replicates to enhance the robustness of the inferred relationships.

Furthermore, linkage disequilibrium (LD) across the avocado genome was assessed by calculating the coefficient of determination (*r*^2^) between pairs of SNP markers using PopLDdecay v. 3.42 [42], with the maximum distance between two SNPs set to 5,000 kb. The LD decay *r*^2^ values were plotted against genetic distance (in kb) using the Perl script Plot_OnePop.pl included with the tool to provide insights into patterns of LD and genetic recombination. The plot was configured with bin1 size set to 1,000, bin2 set to 20,000, and a breakpoint of 10,000 to smooth lines. Finally, ggplot2 in R was used to enhance and finalize the plot.

### Genome-wide association studies (GWAS)

To investigate associations between 4,226 SNP markers and nine fruit morphometric traits across 122 avocado accessions, GWASs were performed with GAPIT (Genomic Association and Prediction Integrated Tool) v. 3.4.0 [43]. Two advanced models were employed: the Fixed and random model Circulating Probability Unification (FarmCPU) model [44] and the Bayesian-information and Linkage disequilibrium Iteratively Nested Keyway (BLINK) model [45]. FarmCPU enhances GWAS by iteratively using associated markers as cofactors in a fixed effect model to control false positives, while avoiding overfitting through a random effect model, resulting in high statistical power and computational efficiency [44]. BLINK eliminates the assumption of even distribution of causal genes required by FarmCPU, working directly on markers instead of bins, and uses the Bayesian Information Content of a fixed effect model for marker selection, further enhancing statistical power and computational efficiency [45].

Additionally, as population structure can introduce confounding and result in false genotype-phenotype associations in GWAS, the top three principal components (PCs) were incorporated as covariates in the analysis to adjust and minimize the effect of population stratification. Significant associations were identified using a strict Bonferroni correction with a significance threshold of -log_10_ (*p*-value) > 4.93. To assess the rate of false positives, the resulting *p*-values were plotted on a Quantile–Quantile (Q-Q) plot by GAPIT to visually compare the observed distribution of *p*-values with the expected distribution under the null hypothesis.

### Genotype-phenotype relationship evaluation

As validation, genotype-phenotype relationships for each identified marker were visualized using box plots depicting phenotypic differences across different genotypes using ggplot2 in R. At the same time, to guide future breeding work, 20 accessions were selected based on fruit weight, width, and diameter − 10 representing the largest accessions and ten the smallest. An allele matrix was constructed using seven selected markers associated with fruit size and weight to illustrate the relationship between these genotypes and fruit size. At the same time, to explore the evolutionary relationships among these accessions and verify the potential relationship between avocado fruit size and the geographical origin of the varieties, a maximum likelihood phylogenetic tree was constructed using Mega 11 [46] with a Tamura-Nei model [47], after alignment on artificial chromosome originating from concatenating 4,226 SNP markers from these accessions using Muscle 5.0 [48]. The topology was supported by 1000 bootstrap replicates.

### GO and KEGG enrichment analyses on candidate genes

Linkage disequilibrium (LD) analysis showed that a 200 kb region, encompassing 100 kb upstream and 100 kb downstream of each marker, corresponds to an approximate *r*^*2*^ value of 0.2. This threshold is considered optimal for capturing genes or genetic regions associated with the identified SNP and the corresponding trait [25, 49]. Based on these analyses, 200-kb windows centered on each identified marker were used to identify potential genes underlying the fruit quality traits. Gene ontology (GO) and Kyoto Encyclopedia of Genes and Genomes (KEGG) enrichment analyses were performed on all candidate genes for fruit sizes (weight, diameter, and width) using Tbtools 2 [50]. The top enriched pathways and components were then visualized in R using ggplot2 with custom code.

## Results

### Phenotypic distribution association analysis

**Fig. 1:**
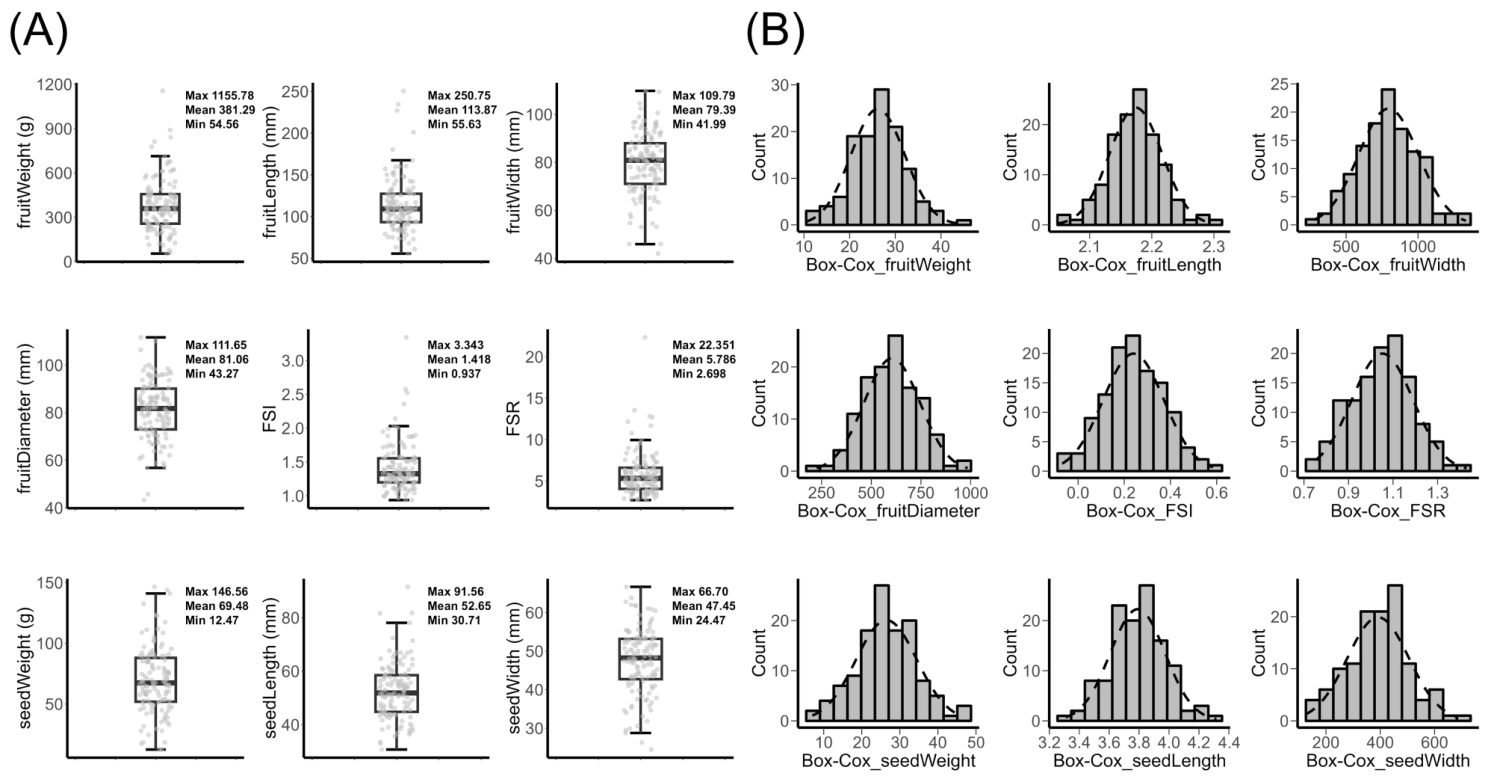
Distribution of nine morphometric traits in 122 avocado accessions. (**A**) Boxplot showing the distribution of original avocado fruit morphometric trait values (**B**) Histogram showing the distribution of avocado fruit morphometric trait values after Box-Cox transformation

The distribution of nine fruit morphometric traits was visualized using box plots, with the mean, minimum, and maximum values labeled on each plot (Fig. 1A). All nine traits underwent Box-Cox transformation to normalize the data, resulting in a well-fitted normal distribution for each trait (Fig. 1B). Among these traits, fruit weight exhibited the broadest range of distribution. The smallest accession, Mexicola, had an average fruit weight of 54.56 g, while the largest accession, Ereguayauin 7, reached 1155.78 g, showing a striking 21.18-fold difference. In contrast, the Fruit Seed Ratio (FSR) had the smallest distribution range, with the largest FSR at 66.7 in Wilson Popenoe, and the smallest FSR at 24.47 in Gainesville, demonstrating a 2.73-fold difference.

Further statistical analysis was performed on nine fruit traits across different ecotype groups as identified in the population analysis conducted in this study (Table 1). It was interesting to note that for all nine traits, including fruit weight and size as well as seed weight and size, the West Indian groups consistently exhibited the highest mean values. The Mexican group, in contrast, showed the lowest mean values, with the Guatemalan group positioned in between. Among these traits, fruit weight displayed the most significant variation between ecotypes. In particular, the average fruit weight of the West Indian group was 432.69 g, which is 1.41 times that of the Guatemalan group at 307.42 g and 2.19 times that of the Mexican group at 197.39 g. Conversely, the Fruit Shape Index (FSI) showed the smallest difference among ecotypes, with values of 1.43 for the West Indian group, and 1.37 for the Mexican group, suggesting a weak or negligible relationship between fruit shape and ecotype.

**Table 1:** Statistical summary of nine fruit morphometric traits across three avocado ecotypes in 122 accessions

Trait	Guatemalan		Mexican		West Indian	
	Mean	SD	Mean	SD	Mean	SD
Fruit Weight	307.42	129.67	197.39	112.07	432.69	171.30
Fruit Length	107.08	24.95	86.76	18.44	120.50	32.65
Fruit Width	75.67	12.38	62.19	12.60	83.46	10.06
Fruit Diameter	76.75	11.09	65.42	12.26	84.79	10.03
FSI	1.40	0.28	1.37	0.18	1.43	0.40
Seed Weight	61.17	27.80	46.48	30.93	75.69	23.31
Seed Length	49.69	9.05	45.28	9.02	54.71	10.37
Seed Width	44.67	9.33	39.27	9.57	49.62	6.76
FSR	5.57	2.21	4.56	1.47	6.07	2.87

Note: Weights are in grams (g) and sizes are in millimeters (mm)

**Fig. 2:**
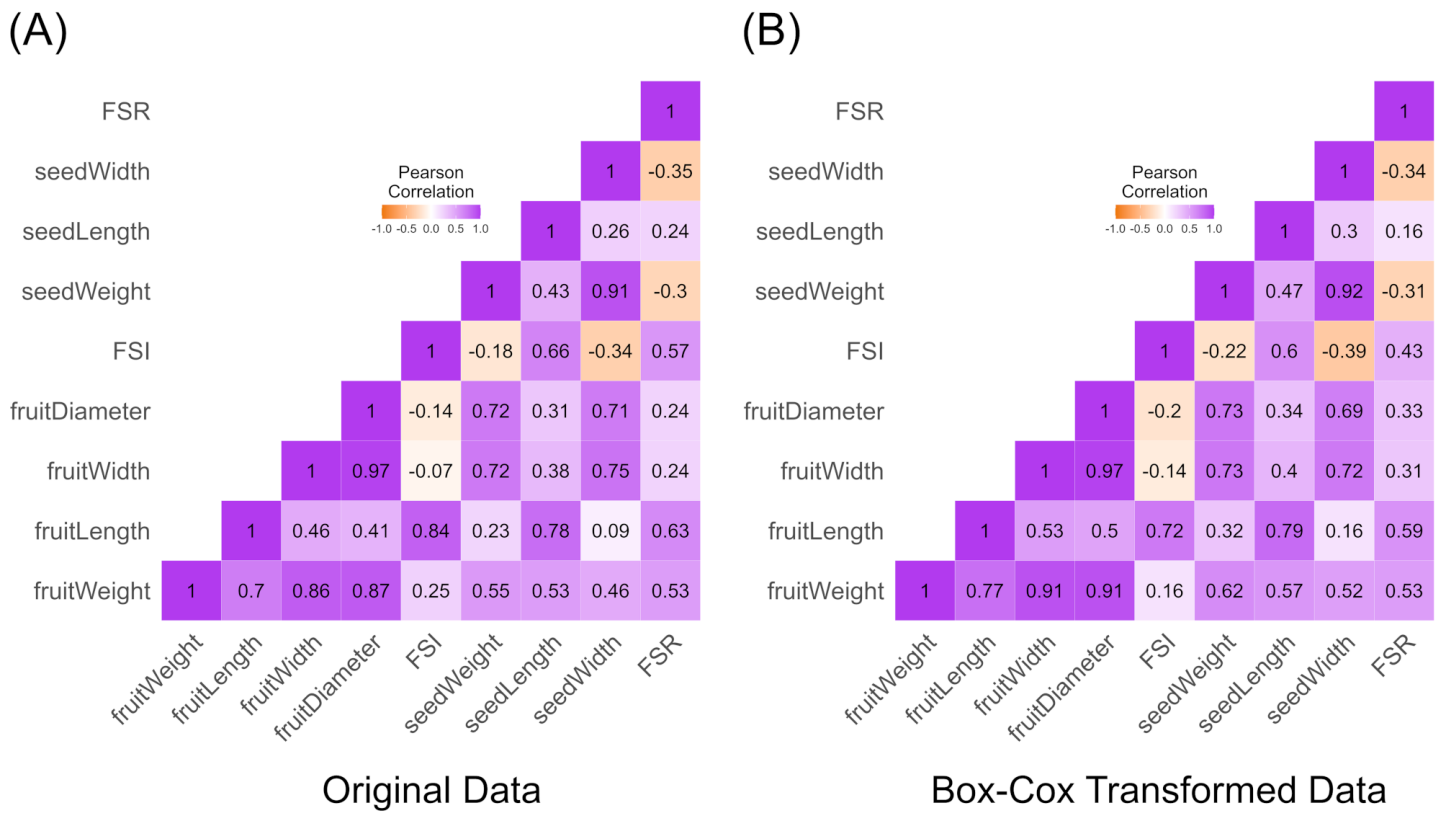
Triangle heatmaps showing Pearson correlations (*r*^2^) between nine fruit morphometric traits in 122 avocado accessions. The strength of correlations is represented by color, with purple indicating a strong positive correlation and orange representing a negative correlation. (**A**) Correlation heatmap for original fruit morphometric data. (**B**) Correlation heatmap for fruit quality traits after Box-Cox transformation

Pearson correlation analysis was conducted to explore relationships between pairs of original fruit traits (Fig. 2A), as well as between pairs of traits after Box-Cox transformation (Fig. 2B). Most fruit size-related traits exhibited strong or moderate correlations. For instance, fruit weight demonstrated very strong correlations with fruit length (*r* = 0.7), width (*r* = 0.86), and diameter (*r* = 0.87). It also showed moderate correlations with seed weight (*r* = 0.55), seed length (*r* = 0.53), seed width (*r* = 0.46), and FSR (*r* = 0.53). As expected, very high correlations were observed between fruit width and fruit diameter (*r* = 0.97), and between seed width and seed weight (*r* = 0.91). Additionally, seed width also strongly correlated with fruit width (*r* = 0.75) and fruit diameter (*r* = 0.71). Conversely, moderate negative correlations were found between FSR and seed weight (*r* = -0.3) and seed width (*r* = -0.35), as well as between FSI and seed width (*r* = -0.34). The two correlation heatmaps generated provided similar results, with only slight differences in coefficient values.

### Population structure and AMOVA analyses

Initially, 5,050 high-quality SNPs were designed, of which 5,025 were retained after mapping to the latest Hass reference genome. After filtering out SNPs with a minor allele frequency (MAF) below 0.05 and those with more than 20% missing data across the accessions, 4,226 SNPs remained. All 122 accessions had a genotype call rate above 95% at these loci, and this dataset was used for further analyses.

**Fig. 3:**
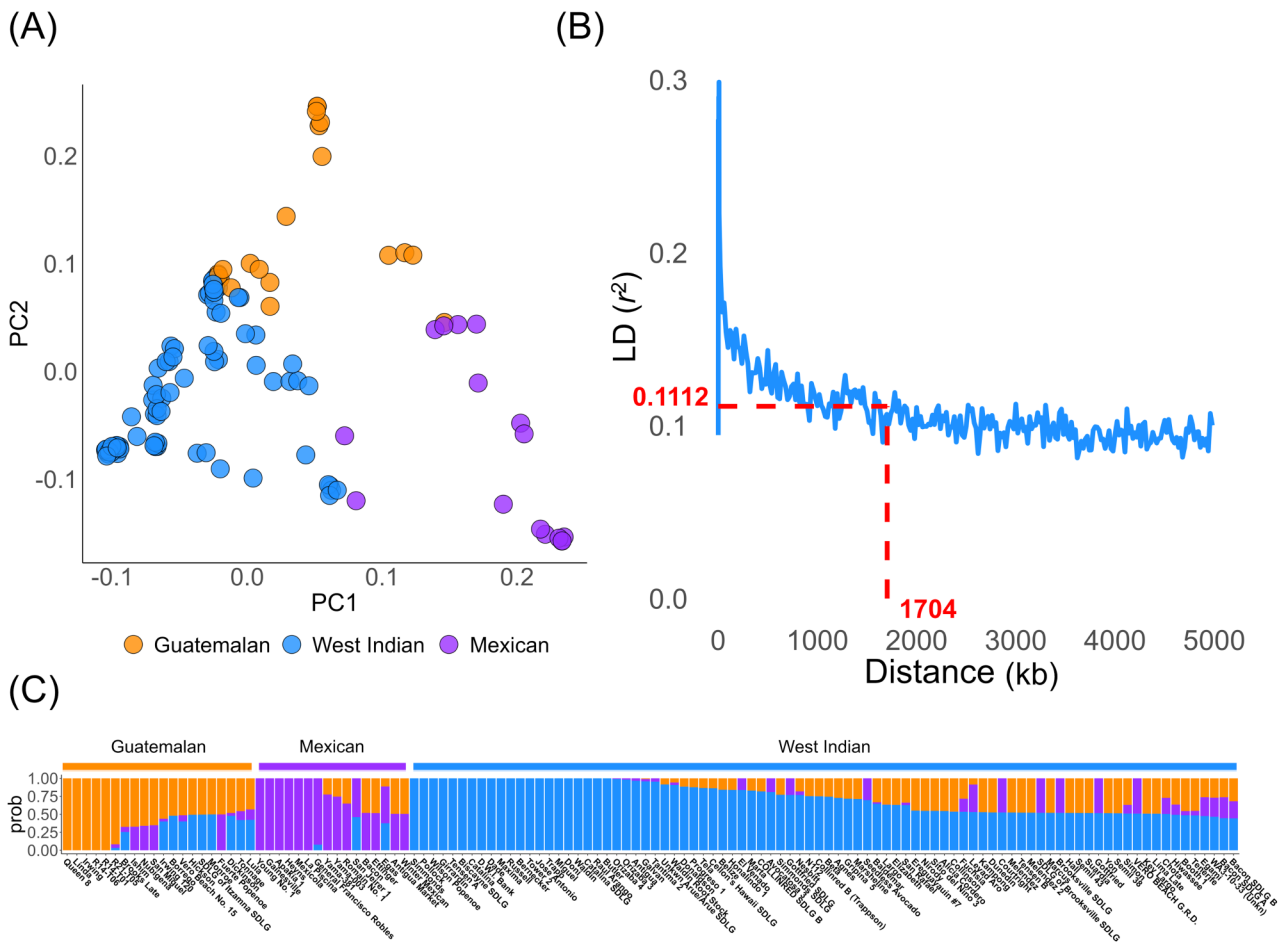
Genetic diversity analysis of 122 avocado accessions using the 4,226 SNP data. (**A**) Principal component plot based on the first two components calculated by PLINK, with each dot representing one avocado accession colored by ecotypes. (**B**) Linkage disequilibrium (LD) decay plot by PopLDdecay, depicting the relationship between LD squared correlation value (*r*^2^) and genetic distance (kb) of SNP pairs. (**C**) Population structure of the 122 avocado accessions when *K* = 3, as determined by ADMIXTURE, where the resulting clusters correspond to three distinct ecotypes of avocados

Linkage disequilibrium was assessed between SNP pairs by plotting the correlation coefficient *r*^2^ against genetic distance (Fig. 3B). The plot revealed a rapid decrease in *r*^2^ with increasing distance between SNPs. On average, the *r*^2^ value was 0.1113 at a genetic distance of 1,704 kb, indicating a low overall linkage disequilibrium. This finding suggests that the high genetic diversity within our collections results in frequent genetic recombination, thereby contributing to the observed low levels of linkage disequilibrium.

Principal component analysis (PCA) based on the first two principal components grouped the 122 avocado accessions into three clusters, corresponding to three ecotypes of avocado (Fig. 3A). Additionally, many hybrids were distributed in the intermediate regions between the clusters.

Based on cross-validation errors, the population structure analysis conducted by ADMIXTURE determined that the optimal number of groups was three (*K* = 3) (Fig. 3C). Each group delineated distinct avocado ecotypes. The first group represented the Guatemalan ecotype and consisted of 20 accessions, accounting for 16.39% of the total accessions. The second group comprised accessions from the Mexican ecotype and contained 16, representing 13.11% of the total accessions. The third group, representing the West Indian ecotype, contained 86 accessions, making up the most significant proportion at 70.49% of the total accessions.

The Analysis of Molecular Variance (AMOVA) revealed that 24% of the genetic variation existed among populations, while 76% occurred within the three populations (Table 2). Further exploration through pairwise PhiPT analysis demonstrated varying levels of genetic differentiation among the avocado ecotypes (Table 2). Specifically, the Guatemalan and West Indian ecotypes exhibited the lowest PhiPT value at 0.164, indicating relatively lower genetic differentiation between these two groups. In contrast, the Mexican and West Indian ecotypes showed the highest PhiPT value at 0.318, suggesting more significant genetic differentiation. The Guatemalan and Mexican ecotypes displayed an intermediate level of genetic differentiation, with a PhiPT value of 0.260.

**Table 2:** AMOVA and pairwise population PhiPT analysis of 122 avocado accessions using 4,226 SNPs

Source	df	SS	MS	Est. Var.	%	Overall PhiPT
Among Pops	2	17084.187	8542.094	274.265	24%	0.241
Within Pops	119	102658.280	862.675	862.675	76%	
Total	121	119742.467		1136.940	100%	
Pairwise Population PhiPT Values						
	Guatemalan	Mexican	West Indian			
Guatemalan	0.000					
Mexican	0.260	0.000				
West Indian	0.164	0.318	0.000			

**Fig. 4:**
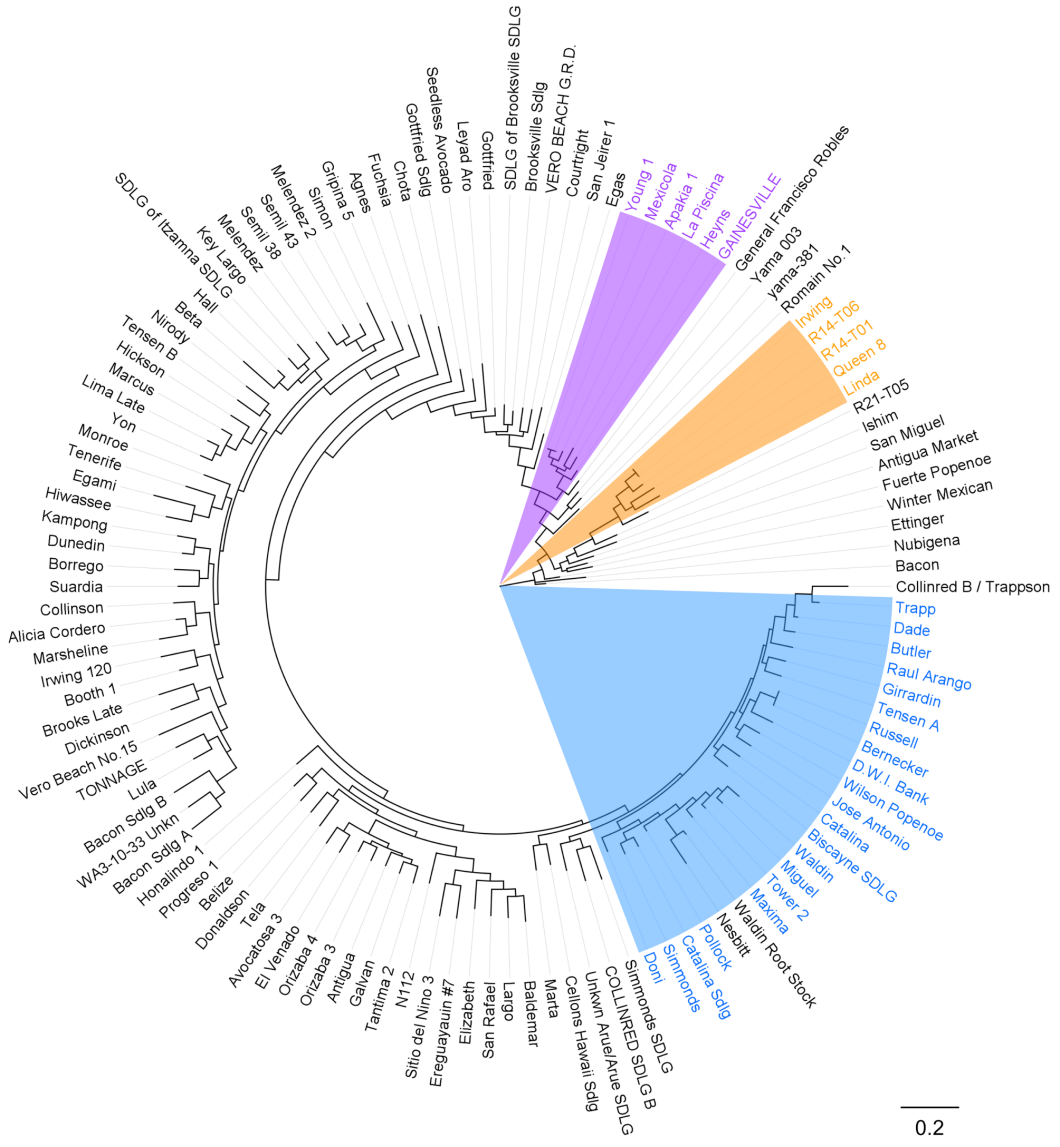
Maximum-likelihood (ML) tree exhibiting phylogenetic relationships among 122 avocado accessions. The tree was constructed using RAxML with the General Time Reversible (GTR) substitution model and gamma-distributed rate heterogeneity. The topology was supported by 1000 bootstrap replicates. Pure lines of the three avocado ecotypes, identified from the population structure analysis, are color-labeled and highlighted (orange: Guatemalan, purple: Mexican, blue: West Indian)

In the phylogenetic tree constructed based on sequence concatenation of 4,226 SNPs, the pure lines from the three ecotypes, identified from the structure analysis, display differing relationships (Fig. 4). Specifically, The Guatemalan and Mexican ecotypes exhibit a closer genetic distance than the West Indian ecotypes. Moreover, the Mexican ecotype appears intermediate between the Guatemalan and West Indian ecotypes in the phylogenetic tree. This discrepancy in phylogenetic relationships among the pure lines of the three ecotypes, compared to the entire population, suggests an overrepresentation of hybrids, particularly the Guatemalan x West Indian hybrids, in our collections, which may have influenced the results.

### Genome-wide association analysis identified key markers associated with avocado fruit morphometric traits

**Fig. 5:**
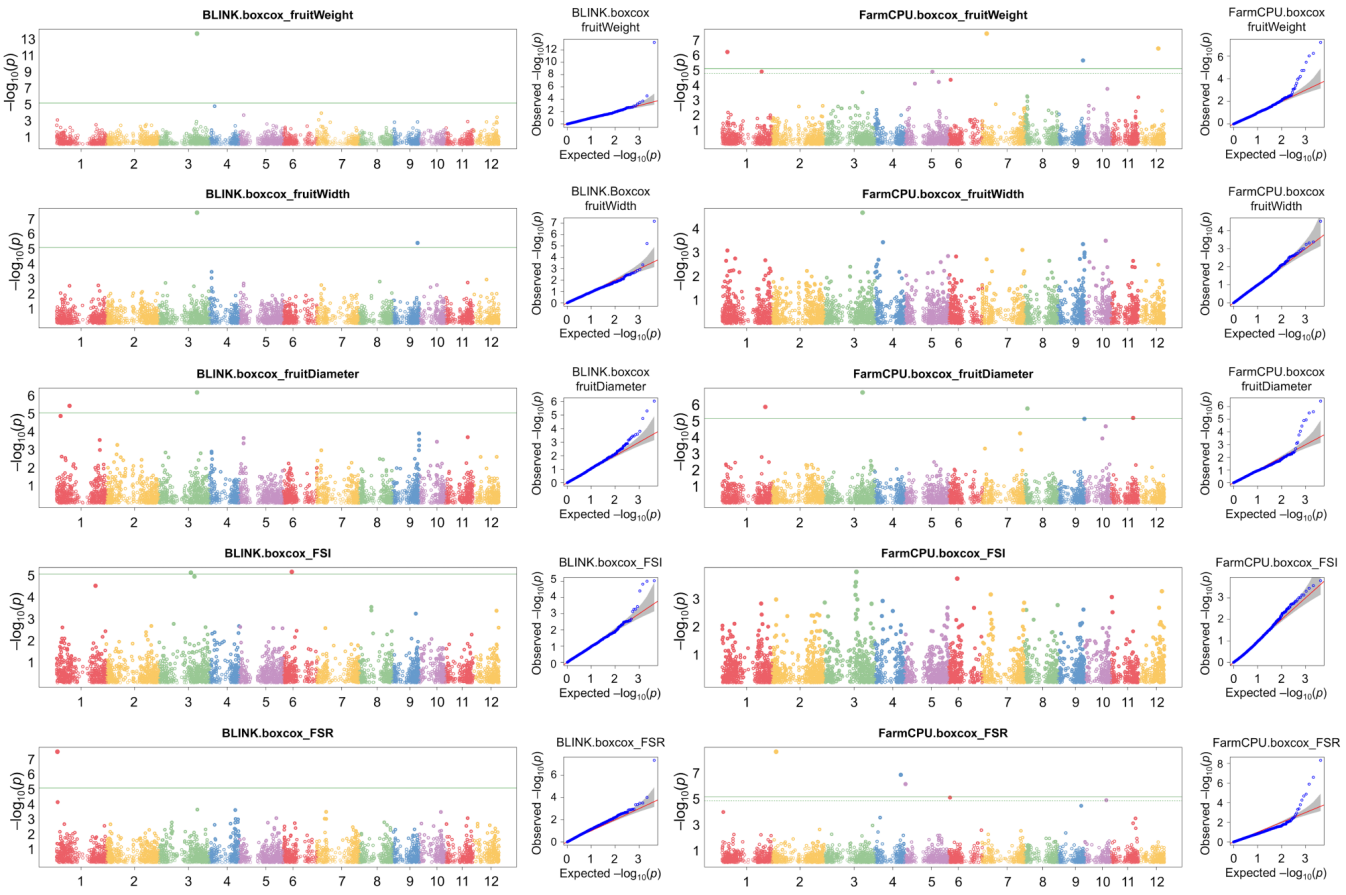
Manhattan and quantile-quantile (Q-Q) plots from GAPIT showing results of GWAS with BLINK and FarmCPU models on avocado morphometric traits. Only traits with significantly associated markers are shown. The x-axis represents the 12 chromosomes of the Hass reference genome (GCA_029852735.1), each colored differently. The y-axis indicates the -log_10_*p* value of the associations. Green horizontal lines show the significance threshold after the Bonferroni correction. In the Q-Q plot, observed -log_10_*(p)* values (y-axis) are compared to expected observed -log_10_*(p)* values (y-axis), with grey shades indicating the 95% confidence interval under the assumption of no association between SNPs and traits

**Fig. 6:**
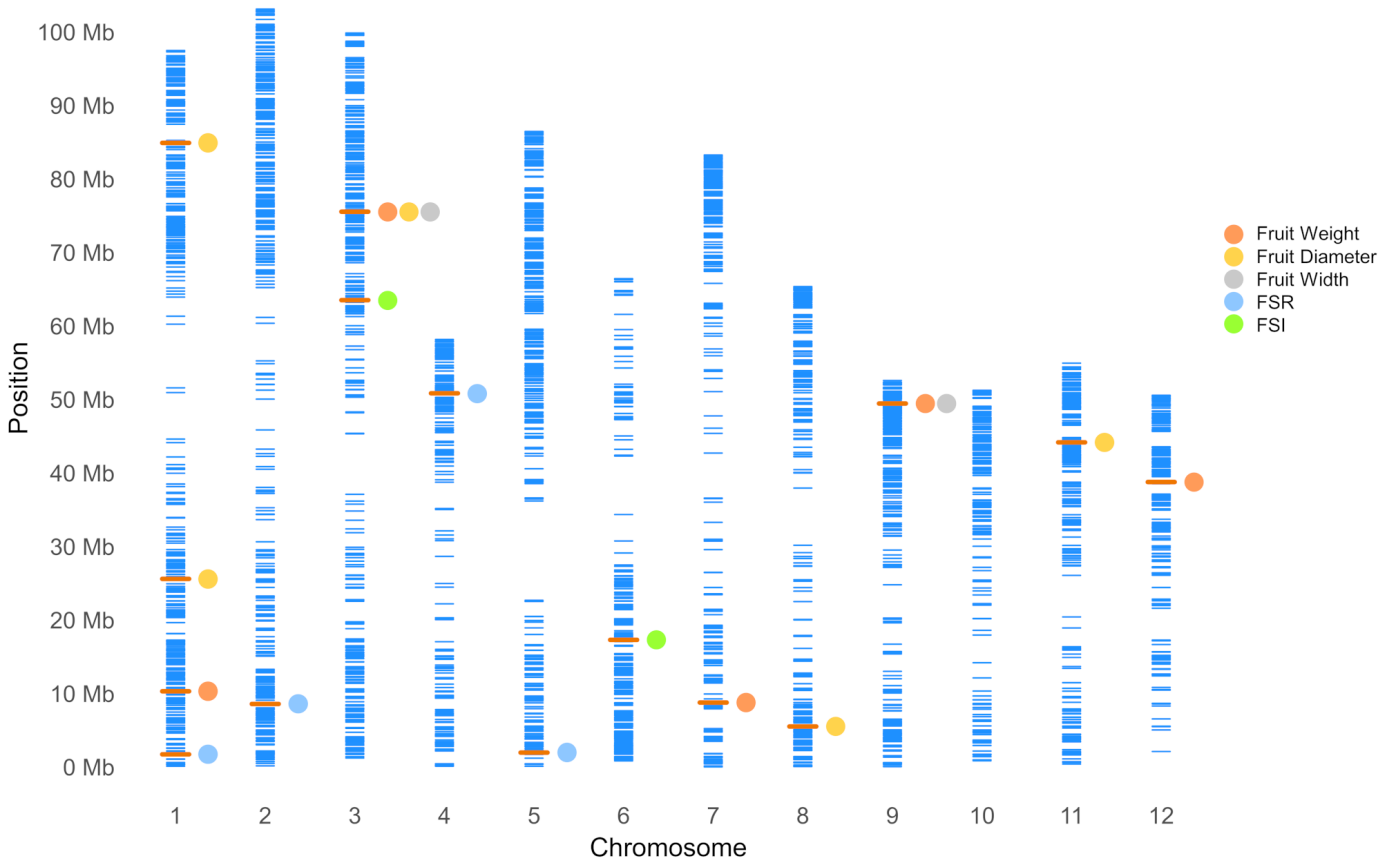
Physical map showing the 4,226 SNP markers on the twelve chromosomes of the Hass genome (GCA_029852735.1). y-axis indicates the physical position on the chromosome in million base pairs (Mb). The x-axis shows the chromosome number. Each blue bar represents one SNP, and the orange bar indicates the position of identified markers in this study. Associated traits are labeled with differently colored dots. Multiple dots in a row indicate common markers associated with multiple traits

GWASs were conducted on nine Box-Cox transformed fruit traits using the FarmCPU and BLINK models. These analyses identified a total of 15 Quantitative Trait Nucleotides (QTNs) with significance exceeding the threshold after Bonferroni correction (− log_10_(*p*-value) = 4.93) (Fig. 5; Table 3). Specifically, BLINK identified 8 QTNs, while FarmCPU identified 11 QTNs. Although FarmCPU identified more QTNs overall, BLINK successfully pinpointed QTNs associated with 5 traits, whereas FarmCPU identified QTNs for only 3 traits. Among these QTNs, five were significantly associated with fruit weight, five with fruit diameter, four with FSR, two with fruit width, and two with FSI. Notably, two common markers were simultaneously associated with multiple traits. Specifically, the marker SHRSPaS002410 showed significant associations with fruit weight, fruit width, and fruit diameter, while SHRSPaS005288 was associated with fruit weight and fruit width employing FarmCPU and BLINK models. The identified QTNs were distributed across all 11 chromosomes, except for chromosome 10 (Fig. 6). Chromosome 1 harbored the most markers, with four QTNs, while chromosome 3 contained two markers. The remaining QTNs were distributed one per chromosome, excluding chromosome 10.

**Table 3:** SNP markers significantly associated with fruit morphometric traits identified in 122 avocado accessions

Trait	SNP	Chr	Position	*p*-value	MAF	Model	SNP Effect	PVE (%)
fruitDiameter	SHRSPaS002410*	3	75,556,021	3.69E-07	0.326446	FarmCPU	-83.7632	
fruitDiameter	SHRSPaS002410*	3	75,556,021	9.2E-07	0.326446	BLINK	-91.0619	49.67708
fruitDiameter	SHRSPaS002896	8	5,529,952	3.17E-06	0.165289	FarmCPU	99.73397	
fruitDiameter	SHRSPaS003688	1	25,607,822	4.91E-06	0.103306	BLINK	84.04543	14.25504
fruitDiameter	SHRSPaS004648	1	84,916,484	2.54E-06	0.322314	FarmCPU	70.34239	
fruitDiameter	SHRSPaS005127	11	44,181,056	1.11E-05	0.404959	FarmCPU	-60.2896	
fruitWeight	SHRSPaS002410*	3	75,556,021	6.02E-14	0.327869	BLINK	-6.33836	60.6479
fruitWeight	SHRSPaS003320	12	38,781,725	5.68E-07	0.122951	FarmCPU	3.160365	
fruitWeight	SHRSPaS004239	1	10,316,058	9.58E-07	0.135246	FarmCPU	2.827334	
fruitWeight	SHRSPaS005288*	9	49,461,432	3.42E-06	0.151639	FarmCPU	2.338652	
fruitWeight	SHRSPaS006540	7	8,786,524	6.09E-08	0.430328	FarmCPU	2.559879	
fruitWidth	SHRSPaS002410*	3	75,556,021	6.66E-08	0.327869	BLINK	-161.622	43.29513
fruitWidth	SHRSPaS005288*	9	49,461,432	6.06E-06	0.151639	BLINK	115.4347	26.84415
FSI	SHRSPaS005460	6	17,311,163	9.26E-06	0.115703	BLINK	-0.0913	20.26087
FSI	SHRSPaS005962	3	63,525,715	1E-05	0.144628	BLINK	0.086007	20.90595
FSR	SHRSPaS002159	4	50,854,429	2.6E-07	0.077869	FarmCPU	-0.10509	
FSR	SHRSPaS002704	5	1,976,337	1.27E-06	0.454918	FarmCPU	-0.05768	
FSR	SHRSPaS006138	1	1,740,883	4.63E-08	0.344262	BLINK	-0.08156	30.33815
FSR	SHRSPaS006582	2	8,592,903	4.78E-09	0.47541	FarmCPU	0.08547	

Asterisks (*) represent common markers associated with multiple traits

Using a 200-kb window, potential genes underlying avocado fruit morphometric traits were identified through functional inference. Marker SHRSPaS002410 was associated with the gene MRB53_011524 for fruit diameter, which encodes the ALTERED XYLOGLUCAN protein involved in cell wall modification [51]. Additionally, SHRSPaS002896 was linked to two candidate genes: the axial regulator *YABBY* (gene *MRB53_025545*) and auxin-induced protein (*MRB53_025547*). The transcription factor gene *MRB53_001570* was connected to SHRSPaS003688. Furthermore, SHRSPaS004648 was associated with the gene *MRB53_003441*, encoding Aux IAA proteins, which act as short-lived transcriptional repressors in the auxin response pathway. Moreover, SHRSPaS005127 was linked to a gene (*MRB53_033740*) in the protein kinase superfamily, suggesting involvement in signal transduction pathways. In the context of fruit weight, SHRSPaS002410 was also identified, highlighting its importance across multiple traits. The transcriptional corepressor gene *MRB53_035549* and the cyclin-dependent kinase inhibitor gene *MRB53_035553* were associated with SHRSPaS003320. Additionally, another transcription factor gene, *MRB53_000750*, was linked to SHRSPaS004239. Furthermore, SHRSPaS005288 was connected to *MRB53_029817*, encoding pectinesterase, an enzyme involved in cell wall modification [52]. Lastly, the protein kinase superfamily gene *MRB53_022336* was associated with SHRSPaS006540. Fruit width also showed connections with SHRSPaS002410 and SHRSPaS005288, associated with the *ALTERED XYLOGLUCAN* gene *MRB53_011524* and pectinesterase gene *MRB53_029817*, respectively. In the context of FSI, SHRSPaS005460 corresponded to *MRB53_020275*, involved in multiple regulatory processes, including ATSRA1, KLK, and PIR. Additionally, SHRSPaS005962 was linked to *MRB53_010821*, encoding the FANTASTIC FOUR protein, which is essential for meristem development [53]. Finally, for FSR, SHRSPaS002159 was associated with the glycosyltransferase 31 family gene *MRB53_014920*, while SHRSPaS006138 and SHRSPaS006582 were connected to ethylene-responsive transcription factor genes *MRB53_000151* and *MRB53_004740*.

### Validation by allele distribution and gene enrichment analyses

**Fig. 7:**
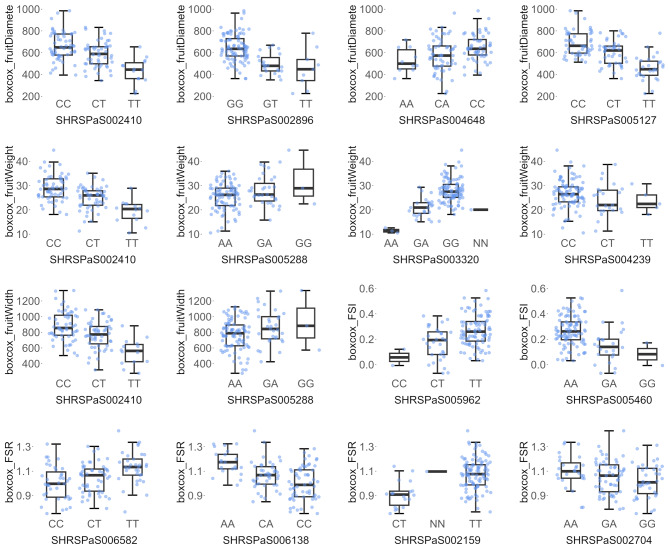
Box plot showing the distribution of Box-Cox transformed fruit traits and their relationship with genotypes for 13 selected markers. The x-axis represents the genotypes showing allele combinations with “NN” as unknown. The y-axis shows the Box-Cox transformed fruit morphometric trait values

The phenotypic variation explained (PVE) by the identified QTNs ranged from 14.26% (SHRSPaS003688 for fruit diameter) to 60.65% (SHRSPaS002410 for fruit weight). Box plots in Fig. 7 illustrate the distribution of phenotypic traits, revealing distinct differences across genotypes. For most traits, the median phenotypic values in accessions homozygous for one allele exceeded those in heterozygous accessions, which, in turn, were higher than the medians in accessions homozygous for the alternate allele. This pattern indicated a strong association between genotype and phenotypic performance.

The QTN SHRSPaS002410 was associated with multiple traits, including fruit diameter, weight, and width. The allele frequency of C at this locus was 67.2%. Among the 56 accessions with homozygous C, the average fruit weight was 469.11 g, the average fruit width was 84.98 mm, and the average fruit diameter was 86.12 mm. These values were significantly higher than the 14 accessions with homozygous T, which had an average fruit weight of 201.64 g, an average fruit width of 63.31 mm, and an average fruit diameter of 65.49 mm.

Another QTN, SHRSPaS005288, was associated with fruit weight and fruit width. The allele G at this locus had a frequency of 15.16%. Although only three accessions out of 122 were homozygous for G, those with allele G (either homozygous or heterozygous) exhibited an average fruit weight of 447.67 g and a fruit width of 83.88 mm. These values were higher than the 88 accessions with homozygous A, which had an average fruit weight of 355.65 g and a fruit width of 77.66 mm.

**Fig. 8:**
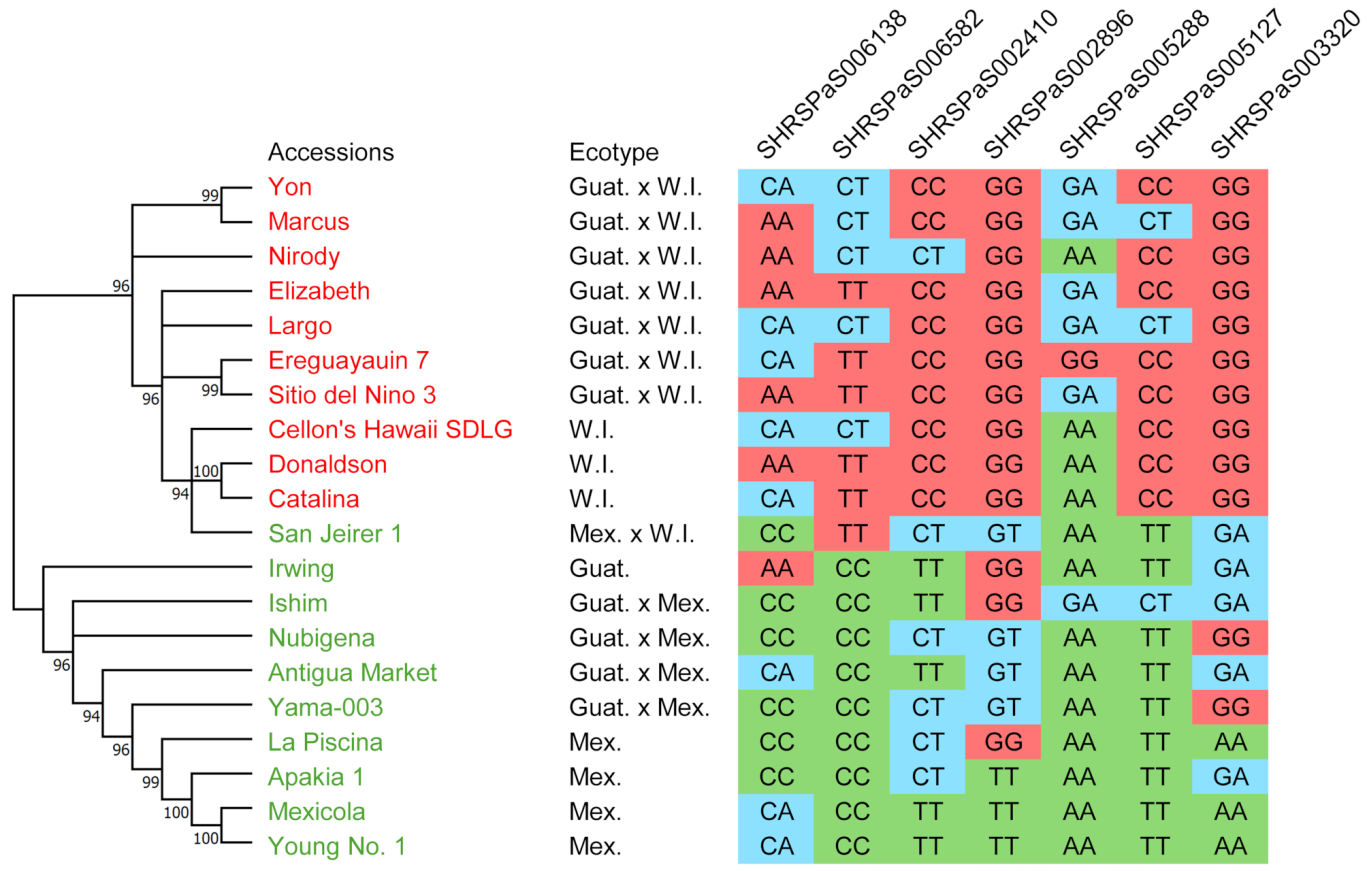
The allele matrix shows allele distribution at 7 SNP loci linked to avocado fruit size, weight, and FSR. Twenty accessions were selected: 10 with the most significant fruits (colored red) and 10 with the most miniature fruits (colored green), based on fruit weight and size data (Supplementary Table S2). A maximum likelihood phylogenetic tree was constructed using the Tamura-Nei model after aligning the concatenated 4,226 SNPs of these 20 accessions with Muscle 5, supported by 1000 bootstrap replicates (bootstrap proportions labeled on branches with a cutoff value of 90) in Mega 11. Ecotypes are labeled after the cultivar names (Guat.: Guatemalan, Mex.: Mexican, W.I.: West Indian, and “x” indicates hybrids between different ecotypes). In the allele matrix, red indicates homozygous alleles for large fruit, green indicates homozygous alleles for small fruit, and blue indicates heterozygous alleles

The top 10 accessions were chosen from both extremes to facilitate future breeding efforts based on fruit weight, width, and diameter (Fig. 8). An allele matrix was drawn for seven chosen QTNs, revealing a clear allele distribution pattern. For example, at QTN SHRSPaS002410, 9 out of 10 accessions with the most significant fruits were homozygous for C, and 1 had a heterozygous CT genotype. Conversely, five accessions with the most miniature fruits were homozygous for T, and another 5 had a heterozygous CT genotype, indicating that allele C was associated with larger fruit size at this locus. A phylogenetic tree was also constructed for these 20 accessions, revealing two clusters. All ten accessions with large fruits were grouped into one cluster, comprising either West Indian ecotypes or hybrids between West Indian and Guatemalan varieties. All accessions with small fruits were grouped into another cluster, except for one hybrid between Mexican and West Indian ecotypes. Notably, 9 were Mexican ecotypes or hybrids between Mexican and other ecotypes, highlighting a solid association between avocado fruit size and geographical origins.

A total of 179 candidate genes (28 for fruit width, 76 for fruit weight, 75 for fruit diameter) were identified within 200 kb windows centered on the 9 QTNs associated with fruit size. These genes underwent Gene Ontology (GO) and Kyoto Encyclopedia of Genes and Genomes (KEGG) enrichment analysis for functional validation.

**Fig. 9:**
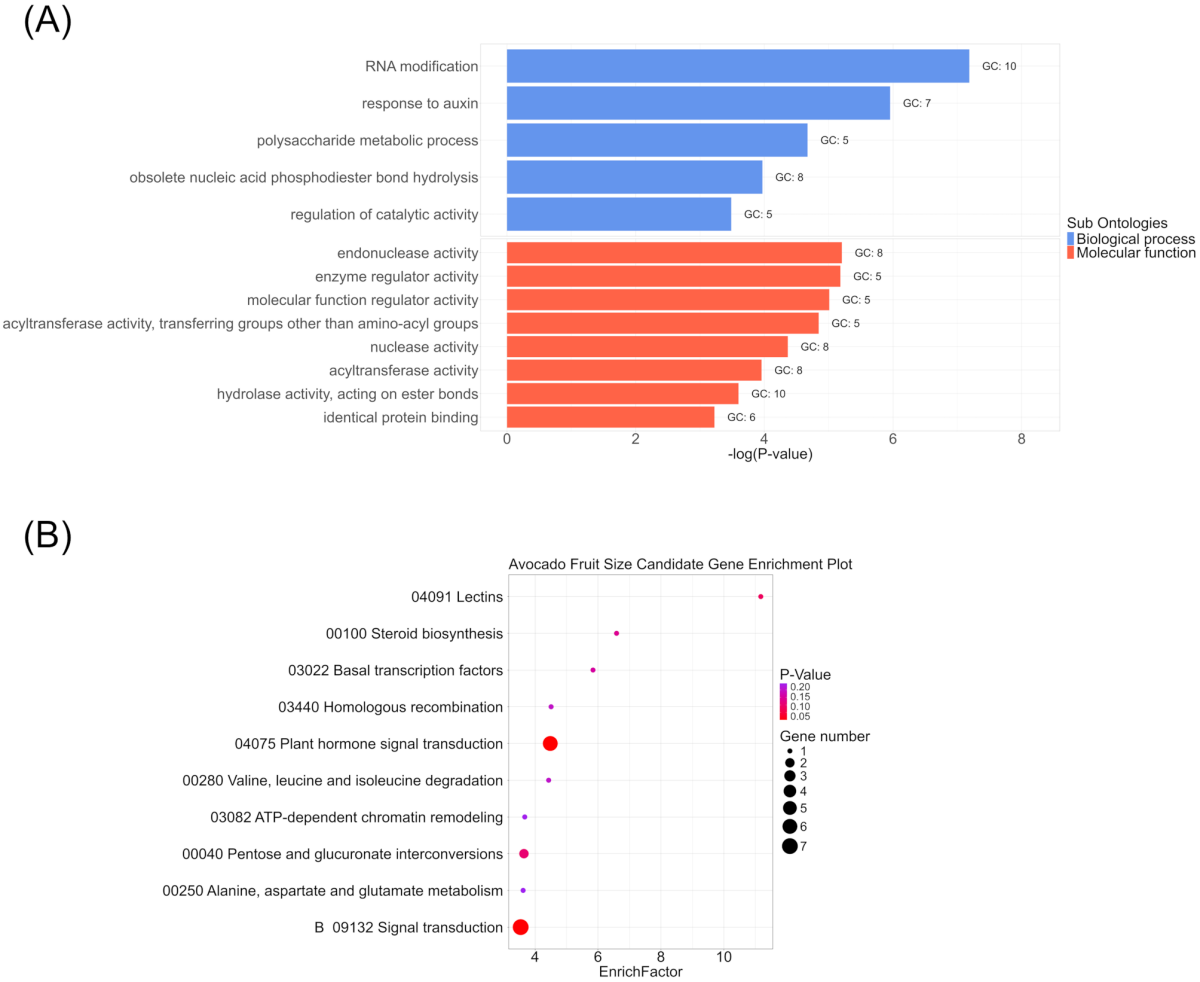
Gene enrichment analyses identify significant pathways associated with fruit size (fruit weight, diameter, width) in avocados. (**A**) Gene Ontology (GO) enrichment analysis identifying top enriched biological processes and molecular functions related to fruit size. (**B**) Kyoto Encyclopedia of Genes and Genomes (KEGG) enrichment analysis showing the top 10 enriched signaling pathways related to fruit size

From GO enrichment analysis, the top enriched biological processes included RNA modification and response to auxin, while the top enriched molecular functions included endonuclease activity and enzyme regulator activity (Fig. 9). Auxin, a class of phytohormones, profoundly influences plant growth and development [54]. It promotes cell division and expansion in tomatoes, strawberries, and other species, ultimately determining fruit size [55–58]. Auxin response factors (*ARF*) are crucial in auxin’s function, as they regulate the expression of auxin-responsive genes. RNA modifications, such as N4-acetylcytidine (*ac*^*4*^*C*) in tomato mRNA, have been linked to fruit ripening and ethylene signaling, influencing fruit development and ripening [59]. Enzyme regulators, such as 3-hydroxy-3-methylglutaryl coenzyme A reductase (*HMGR*) and the sucrose non-fermenting 1-related protein kinase (*SnRK1*) complex, play roles in early cell division, impacting fruit size in litchi [60], and Hass avocado [61, 62]. Endonucleases are crucial for maintaining genomic stability and regulating cell growth, which play essential roles in fruit development [63].

The KEGG pathways enrichment analysis revealed two significantly enriched pathways: plant hormone signal and transduction and (general) signal transduction (Fig. 9). Plant hormones are crucial for regulating plant and fruit growth and development, and their signal transduction pathways are vital for determining fruit size. Beyond auxin, other hormones such as gibberellin, ethylene, abscisic acid, and cytokinin are also involved in fruit development [20, 56, 57]. The broader concepts of signal transduction encompass general cell responses to various signals, including those that regulate plant and fruit growth and ultimately impact fruit size.

## Discussion

In this study, a comprehensive genetic diversity analysis based on 4,226 SNP markers was performed on 122 selected avocado accessions growing in the USDA ARS SHRS field. The population structure and principal component analyses divided the 122 accessions into three groups, consistent with the three ecotypes of avocados. The population pairwise PhiPT analysis indicated a close relationship between the West Indian and Guatemalan ecotypes compared to the Mexican group in the 122 selected accessions. However, the phylogenetic tree constructed on 4,226 SNP markers demonstrated a close distance between the pure lines of Guatemalan and Mexican ecotypes than West Indian, aligning with the previous studies [64–67]. The potential explanation is that the overrepresentation of Guatemalan x West Indian hybrids in our West Indian group significantly impacted the result by reducing genetic differentiation between the two groups. This is consistent with the previous report that the West Indian and Guatemalan x West Indian hybrids dominate cultivars in Florida [8].

The observed patterns in the statistical analyses of phenotypic data across the three different ecotype groups highlight intriguing insights into the fruit morphometric traits of avocados. Specifically, the consistent trend of higher mean values in the West Indian groups compared to the Guatemalan group, and subsequently the Mexican groups, across various traits like fruit weight, length, width, diameter, seed weight, seed length, seed width, fruit shape index (FSI), and fruit seed ratio (FSR), suggests potential differences in fruit development and composition among these ecotypes. Notably, the most substantial disparity was evident in average fruit weight, with the West Indian group exhibiting a weight 2.19 times as much as the Mexican group. These findings resonate with earlier morphological records documenting distinctions among the three ecotypes of avocados [5, 68]. This discussion prompts further exploration into the underlying genetic and physiological mechanisms contributing to these observed differences, which could inform targeted breeding strategies to enhance fruit quality and productivity in avocado cultivars. Correlation analyses revealed that most fruit traits, especially those related to weight and size, are strongly correlated. Sizing rings and calipers have both been widely used in fruit phenotyping [69]. Fruit diameter is calculated using a sizing ring, which measures the largest circumference of the fruit. In contrast, fruit width is the horizontal distance measured directly using calipers. These two measurements, while similar, capture distinct aspects of fruit morphology. Different genetic factors might influence these dimensions, and analyzing both can help identify specific genetic markers associated with each trait.

GWAS was conducted to identify potential genotype-phenotype associations for nine fruit morphometric traits, utilizing 4,225 SNPs from 122 avocado accessions. Fifteen significant markers were identified, distributed across all chromosomes except chromosome 10, linked to five fruit quality traits, including fruit weight, diameter, width, shape index (FSI), and fruit seed ratio (FSR). The most significant marker, SHRSPaS002410, was found to be associated with multiple fruit morphometric traits, specifically fruit weight, fruit width, and fruit diameter. A gene encoding the protein ALTERED XYLOGLUCAN (MRB53_011524) was identified within this genomic region. The xyloglucan network, regulated by the xyloglucan endotransglucosylase/hydrolase (*XTH*) gene, plays a pivotal role in fruit development. Studies in transgenic tomatoes have shown that elevated levels of *SlXTH1* transcripts are positively correlated with larger fruit size, while suppressed *SlXTH1* expression leads to smaller fruit [70]. The mechanism underlying this involves xyloglucan endotransglycosylase (*XET*) activity, which peaks during early fruitlet stages and decreases as fruit expands [71]. *XET* promotes cell expansion in rapidly growing fruit cells by temporarily loosening cell walls [51]. Whether these genes are involved with fruit size will require further studies including high-resolution SNP markers alongside functional analyses, which are beyond the scope of this report.

Another significant marker, SHRSPaS005288, associated with fruit weight and width, was linked to the gene *MRB53_029817*, encoding pectinesterase. Pectinesterase plays a crucial role in fruit development and ripening by modifying the cell wall through the breakdown of pectin, a significant component of the plant cell wall. This process is essential for plant cell division and fruit expansion [72]. Additionally, increased pectinase activity during fruit ripening leads to fruit softening, changes in water content, and changes in fruit texture [73]. The solubilization of pectin is a common characteristic in fruit ripening, and this is evident in avocados [74], possibly facilitated by the action of pectinase enzymes [73].

Marker SHRSPaS002896, associated with fruit diameter, was linked to the gene *MRB53_025545*, which encodes the axial regulator *YABBY*. This gene plays a crucial role in plant organ development. For instance, in tomatoes, the *YABBY* gene family member *SlYABBY2a* exhibits specific expression in the fruit septum. Mutations leading to *SlYABBY2a* deficiency result in altered fruit shape characterized by inward concavity in the pericarp [75]. Marker SHRSPaS002896 is also associated with the gene *MRB53_025547*, which encodes an auxin-induced protein. Auxin-induced proteins play significant roles in various plant growth and developmental processes by responding to auxin, an essential plant hormone regulating cell elongation, division, and differentiation [54, 76]. Another significant marker, SHRSPaS004648, also associated with fruit diameter, is related to the gene *MRB53_003441*, encoding Aux IAA proteins. These proteins are short-lived transcription factors acting as repressors to inhibit the function of auxin response genes in the early stages when auxin concentrations are low, thereby ensuring precise control of overgrowth and developmental processes [77].

Furthermore, two markers, SHRSPaS004239 and SHRSPaS003320, associated with fruit weight, are also related to transcription-regulating elements genes *MRB53_000750* and *MRB53_035549*, although their specific functions remain unclear. While these genes may not directly determine fruit size, they could indirectly influence it by regulating the expression of other genes involved in fruit growth and metabolism. Similarly, two genes, *MRB53_022336* at SHRSPaS006540 and *MRB53_033740* at SHRSPaS005127, both from the protein kinase superfamily and with unknown function, may regulate signaling pathways affecting processes such as cell division, cell expansion, hormone signaling, and metabolism to impact fruit size indirectly [78, 79].

Marker SHRSPaS005962, associated with FSI, is related to the gene *MRB53_010821*, encoding protein FANTASTIC FOUR (FAF). *FAF* is known for its role in regulating flowering time in tomatoes, and transgenic lines with overexpression of *SlFAF1/2a* and *SlFAF1/2c* have shown altered fruit size, resulting in smaller fruits than the wild type [80]. FAF’s orthologous gene CELL SIZE REGULATOR (CSR) is also expressed in fruit tissues during maturation, increasing mesocarp cell size and, consequently, larger fruit in tomatoes [81].

Two markers, SHRSPaS006138 and SHRSPaS006582, associated with FSR, are related to genes *MRB53_000151* and *MRB53_004740*; both encode ethylene-responsive transcription factors. Ethylene is a key phytohormone regulating fruit ripening and inducing various physiological and biochemical changes, including cell wall softening, chlorophyll degradation, and starch-to-sugar conversion, influencing fruit texture, flavor, and overall weight [82]. Moreover, ethylene also regulates seed development, as seen in loss-of-function mutants like *SlEIN2-1*, which exhibit decreased auxin biosynthesis and result in smaller fruits with reduced seed numbers [83]. Ethylene signaling pathways may intricately control the overall fruit-to-seed ratio by modulating fruit and seed development.

## Conclusions

The study has successfully identified markers significantly associated with crucial avocado fruit morphometric traits, including fruit weight, size, fruit shape index, and fruit seed ratio. It extends previous research on fruit color, shape, skin texture, and flavor [84]. These findings promise to advance avocado breeding efforts by facilitating marker-assisted selection in developing elite cultivars. Candidate genes linked to these genetic regions have been pinpointed, shedding light on the potential mechanisms governing these traits. The candidate genes influencing fruit size can be broadly categorized into two groups: those regulating the plant hormone pathway (specifically auxin and ethylene) and those directly modifying the cell wall during early fruit development, thereby promoting cell expansion. Future research endeavors aim to delve deeper into these mechanisms by fine-mapping polymorphisms from high-resolution GBS data to precisely locate the functional gene regions. Additionally, transcriptome analyses will be conducted to validate gene expression levels and understand their functional significance. Further validation includes gene editing techniques such as CRISPR-Cas to manipulate gene function and assess the resulting plant phenotypes in gain- and loss-of-function mutants [85]. These comprehensive approaches will significantly enhance our understanding of the mechanisms underlying fruit size determination in avocados, ultimately aiding in developing superior avocado cultivars with desired fruit characteristics.

## Electronic supplementary material

Below is the link to the electronic supplementary material.

Supplementary Material 1

## Data Availability

Data is provided within the manuscript or supplementary information files.
