# Correction: Genome-wide association study identifies key quantitative trait loci (QTL) for fruit morphometric traits in avocado (*Persea spp.*)

**DOI:** 10.1186/s12864-024-11134-z

**Published:** 2024-12-11

**Authors:** Jin Li, Shamseldeen Eltaher, Barbie Freeman, Sukhwinder Singh, Gul Shad Ali

**Affiliations:** 1 United States Department of Agriculture, Agricultural Research Service, Subtropical Horticulture Research Station, Miami, FL USA; 2 Present address: Department of Plant Biotechnology, Genetic Engineering and Biotechnology Research Institute (GEBRI), University of Sadat City, Sadat, Egypt

**Correction: BMC Genomics 25, 1135 (2024)**

**https://doi.org/10.1186/s12864-024-11043-1**

Following publication of the original article [1] it was reported that Fig. 5 and Fig. 6 were transposed. The figure captions were correct, but the images were swapped.

**Fig. 5:**
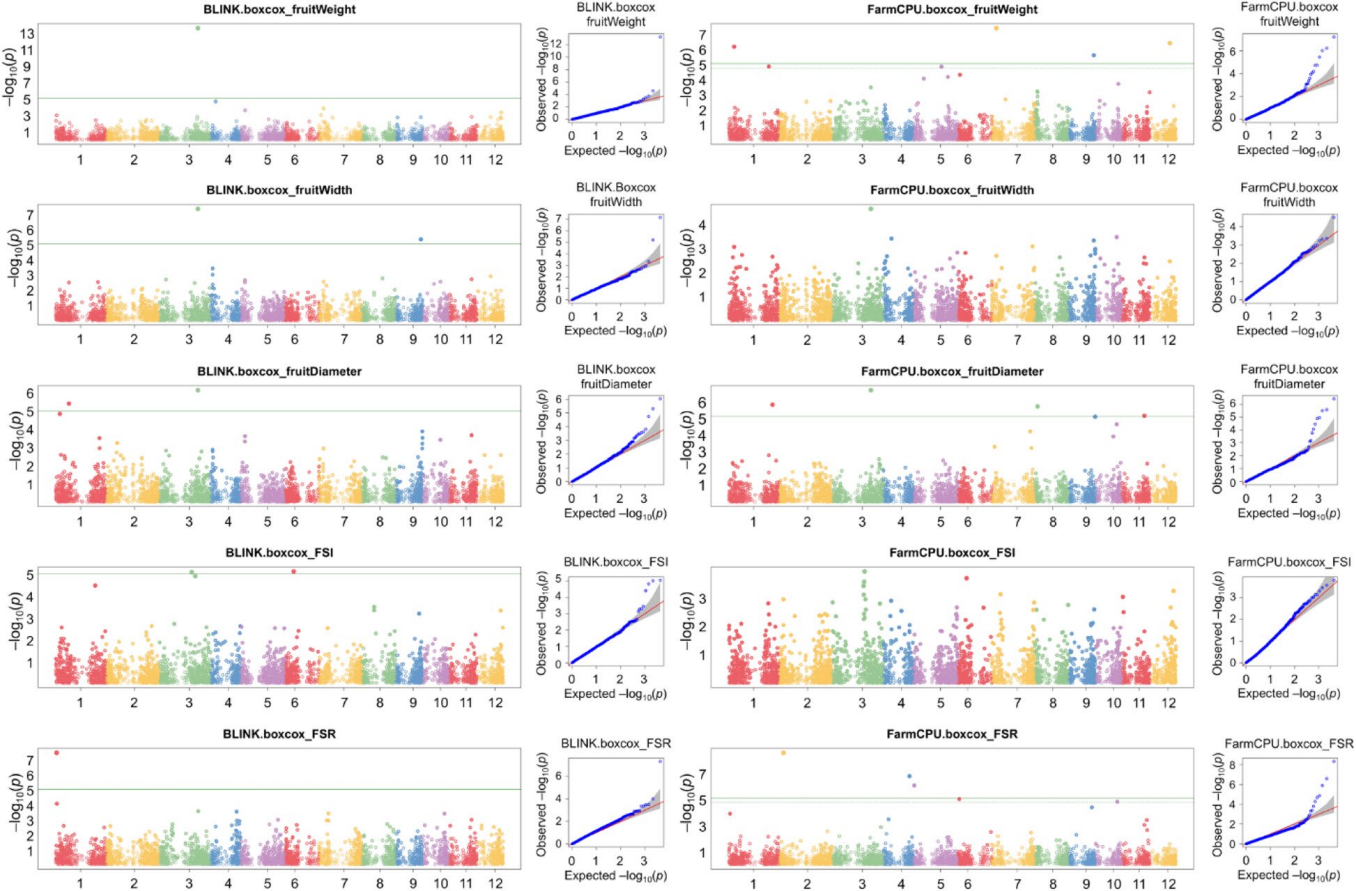
Manhattan and quantile–quantile (Q-Q) plots from GAPIT showing results of GWAS with BLINK and FarmCPU models on avocado morphometric traits. Only traits with significantly associated markers are shown. The x-axis represents the 12 chromosomes of the Hass reference genome (GCA_029852735.1), each colored differently. The y-axis indicates the -log_10_*p* value of the associations. Green horizontal lines show the significance threshold after the Bonferroni correction. In the Q-Q plot, observed -log_10_*(p)* values (y-axis) are compared to expected observed -log_10_*(p)* values (y-axis), with grey shades indicating the 95% confidence interval under the assumption of no association between SNPs and traits

**Fig. 6:**
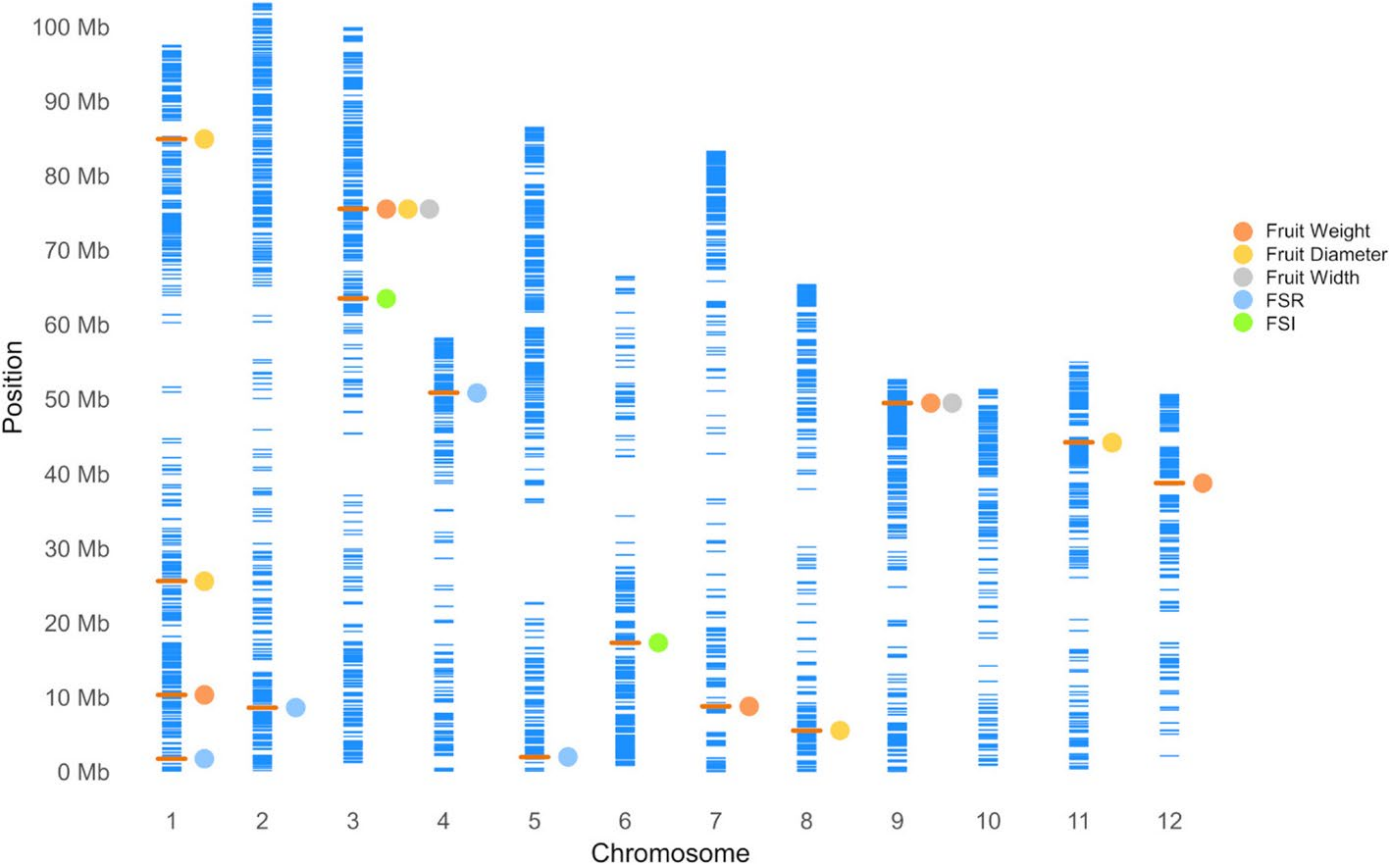
Physical map showing the 4,226 SNP markers on the twelve chromosomes of the Hass genome (GCA_029852735.1). y-axis indicates the physical position on the chromosome in million base pairs (Mb). The x-axis shows the chromosome number. Each blue bar represents one SNP, and the orange bar indicates the position of identified markers in this study. Associated traits are labeled with differently colored dots. Multiple dots in a row indicate common markers associated with multiple traits

The figures with their correct captions are given in this Correction, and the original article has been updated.
